# Long-Term Follow-Up of Biological Reconstruction with Free Fibular Graft after Resection of Extremity Diaphyseal Bone Tumors

**DOI:** 10.3390/jcm11237225

**Published:** 2022-12-05

**Authors:** Zhaohui Li, Zhen Pan, Hua Guo, Xiang Fei, Dongdong Cheng, Qingcheng Yang

**Affiliations:** Department of Orthopedics, Shanghai Sixth People’s Hospital Affiliated to Shanghai Jiao Tong University School of Medicine, Shanghai 200233, China

**Keywords:** free fibular graft, autograft, allograft, diaphyseal reconstruction, bone tumor

## Abstract

This study aimed to evaluate the clinical outcomes and complications of reconstruction with a composite free fibula inside other biological grafts. We retrospectively reviewed 26 patients who underwent reconstruction after bone tumor resection of the diaphysis of the long bone. Surgical data, time to bony union, functional outcomes, and complications were evaluated in all cases. The median follow-up was 72.5 months. The limb salvage rate was 100%. Primary osseous union was achieved in 90.4% of the junctions. The union rates at the metaphyseal and diaphyseal junctions were 100% and 85.7%, respectively (*p* = 0.255). The mean time of bony union in the upper (87.5%) and lower (91.7%) extremity was 4.6 ± 1.6 months and 6.9 ± 2 months, respectively. The mean MSTS score was 27.2 ± 3.2, with a mean MSTS rating of 90.7%. Complications occurred in 15.4% of the cases. The administration of vascularized or non-vascularized grafts did not significantly influence the union time (*p* = 0.875), functional outcome (*p* = 0.501), or blood loss (*p* = 0.189), but showed differences in operation time (*p* = 0.012) in lower extremity reconstruction. A composite free fibula inside other biological grafts provides a reasonable and durable option for osseous oncologic reconstruction of the long bone diaphysis of the extremities with an acceptable rate of complications. A higher union rate was achieved after secondary bone grafting. In lower-extremity reconstruction, two plates may be considered a better option for internal fixation. Vascularizing the fibula did not significantly affect the union time.

## 1. Introduction

With the rapid development of imaging, adjuvant chemotherapy, and surgical technology, amputation has been routinely replaced by limb salvage, resulting in higher patient satisfaction following the surgical treatment of bone tumor [1]. Limb function is preserved to the maximum extent without decreasing the long-term survival rate of bone tumor patients [2,3]. Limb salvage surgery must strictly abide by the golden surgical principle––the tumor-free principle––which results in massive bone defects following tumor resection. Reconstruction after tumor resection is a challenging problem that has become a hot topic for orthopedic oncologists. Successful recovery of limb function depends on the long-term stability of the reconstruction after resection of a diaphyseal bone tumor. In addition, postoperative complications, such as infection, nonunion, and graft loosening, are major challenges in the reconstruction of bone defects.

Currently, the commonly used surgical reconstructive options can be divided into biological and non-biological reconstruction. The former includes allograft reconstruction, free fibular graft reconstruction, and extracorporeal devitalized autograft reconstruction, while the latter mainly includes segmental prosthesis and bone cement reconstruction. All reconstruction procedures have advantages and disadvantages. Thus, the strategy to apply a reconstructive graft relies on a comprehensive consideration of the patient’s individual characteristics. Compared to non-biological reconstruction, biological reconstruction can improve long-term function [4].

Since Weland et al. reported the first free-vascularized fibular graft for long bone reconstruction after resection of a bone tumor in 1977 [5], the free-vascularized fibular graft has become a popular option in limb-salvage surgery [6,7]. Non-vascularized fibular grafts and free-vascularized fibular grafts are the two forms of fibula used by orthopedic surgeons. Previous studies have shown that vascularized fibular grafts can mimic the process of bone healing after fracture and enhance biological union rather than creeping substitution [6,7]. As described above, free-vascularized fibular grafts appear to be a more reliable option. It has been reported that free-vascularized fibular grafts can achieve higher union rates than fibular grafts without vascularization in the reconstruction of long bone defects [8,9]. However, comparing between the two forms of fibula, the use of non-vascularized fibular grafts results in a shorter and simpler procedure. A recent study that evaluated the clinical outcomes of fibular grafts in the reconstruction of bone defects showed that there was no significant difference in graft hypertrophy and functional outcomes between vascularized and non-vascularized fibular grafts [10]. Ogura et al. [11] conducted a retrospective review of 11 patients and reported a satisfactory outcome following the use of vascularized fibulas in the reconstruction of the lower extremity. A systematic review of the literature indicated that free-vascularized fibular grafts are an effective treatment strategy for upper extremity bone sarcomas. However, the authors also noted a relatively high rate of postoperative complications [12]. Reported postoperative complications include fatigue fracture of the graft, delayed union, nonunion, infection, and donor site complications [4,5,9]. In addition, the diameter of the fibular graft may not match the defect site, especially in the lower limb, which makes it difficult for the graft to restore the original mechanical structure and even increases the probability of complications and reconstruction failure.

Here, we present the findings of 26 patients who underwent fibular graft reconstruction combined with other biological grafts, following wide resection of diaphyseal tumors in the extremities. We also discuss the radiological and clinical outcomes associated with this technique. We aimed to contribute to finding the most clinically useful procedure to reconstruct bone defects after the resection of extremity diaphyseal bone tumors.

## 2. Materials and Methods

### 2.1. Patients

We conducted a retrospective review of 26 patients who underwent resection of an extremity diaphyseal bone tumor and reconstruction using a fibular graft combined with an extracorporeal devitalized autograft or a massive bone allograft between September 2011 and December 2018. There were 14 female and 12 male patients with a mean age of 42.0 ± 16.7 years at the time of operation. Bone tumors included osteosarcoma (*n* = 7), adamantinoma (*n* = 3), osteofibrous dysplasia (*n* = 3), Ewing’s sarcoma ( *n* = 2), hemangioma of bone (*n* = 1), and chondrosarcoma (*n* = 1), and bone metastases (*n* = 9, four cases of renal cell cancer, three cases of thyroid carcinoma, and two cases of bladder cancer). The tumor sites were the humeral diaphysis (*n* = 8), tibial diaphysis (*n* = 12), and femoral diaphysis (*n* = 6) (Table 1).

### 2.2. Surgical Technique

**Figure 1:**
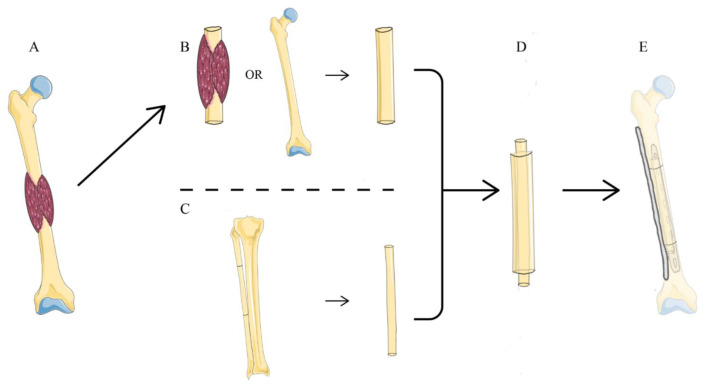
Diagram showing biological reconstruction methods after resection of diaphyseal bone tumor in the femur. (**A**) The tumor is located in the diaphysis of femur. (**B**) Preparation of devitalized autograft or allograft. (**C**) Preparation of the ipsilateral fibular graft. (**D**) The fibula is inserted into the intramedullary canal of devitalized autograft or allograft. (**E**) Reconstruction of the diaphyseal defects with the fibular graft using double plates for internal fixation.

**Figure 2:**
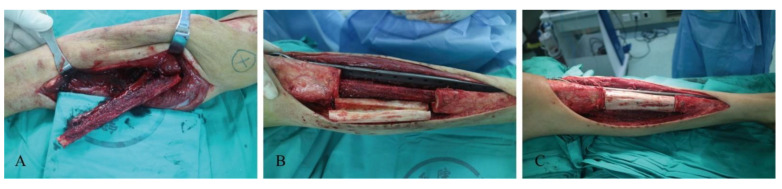
Photographs of the main procedures of reconstruction in the tibia. (**A**) Preparation of the ipsilateral fibular graft. (**B**) Replantation of devitalized autograft. (**C**) Assembly and fixation of devitalized autograft and fibular graft.

All the patients underwent preoperative radiography to determine the anatomy of the operative area. In total, 25 patients underwent extremity reconstruction with simultaneous tumor resection. One patient was diagnosed with prosthesis failure after tumor resection, and reconstruction was performed concurrent with the removal of the previously failed prosthesis. The case of hemangioma included in our series is a low-grade malignant vascular tumor, not a simple hemangioma of bone, so we also took a segmental resection of the tumor. As for the cases of osteofibrous dysplasia included in our study, we considered the possibility of fibrous lesion malignancy before surgery. The standard approach is to resect tumors with wide margins (at least 2 cm). The extent of resection did not involve the metaphyseal plate in case 24, who was eight years old. In most cases, the ipsilateral fibula was harvested for reconstruction, allowing for easier postoperative mobilization. The length of the fibula was determined on the basis of the defect size. For fibular osteotomy, a sufficient distal fibula was maintained to prevent ankle instability. The fibula was then passed into the medullary canal of the extracorporeal devitalized autograft (*n* = 21) or the massive bone allograft (*n* = 5). During the procedure of devitalization, the tumor-bearing bone was immerged into 75 % alcohol for 30 min, then retrieved and flushed with saline. The composite reconstruction graft was fixed to the host bone via internal fixation using plates. The non-vascularized fibulas were used in 12 cases. In particular, the vascular pedicle of the graft was anastomosed to a branch of the artery when a vascularized fibula was utilized. In tibial cases, the pedicled vascularized fibula harvested in the ipsilateral leg was used as a vascularized graft for its comparative technical ease, and only one free vascularized fibula was harvested from the opposite limb of the recipient bone, because the ipsilateral fibula was removed. For upper extremity reconstruction, the patients had their upper limbs immobilized for at least 4 weeks. For lower extremity reconstruction, partial weight-bearing walking with crutches was allowed 6–8 weeks after surgery, and full weight-bearing walking was allowed 6–12 months postoperatively. A schematic diagram and series of surgical photographs were used to illustrate the surgical procedures mentioned above (Figure 1 and Figure 2).

### 2.3. Follow-Up

Patients were assessed clinically and radiologically by the same surgeon. During follow-up, radiographs were reviewed by the surgeon to assess the evidence of union. Bone union after surgery was defined as the presence of an indistinct or absent osteotomy line or callus formation. The time of bony union was recorded. Functional outcomes were assessed by using the MSTS scoring system. Postoperative complications were recorded. The median follow-up of the patients was 72.5 months (37–124 months).

### 2.4. Statistical Analysis

Data were analyzed using SPSS version 26.0 (IBM Corp, Armonk, NY, USA). Continuous variables are reported as the mean ± standard deviation. The length of follow-up is expressed as median and ranges. Comparisons between different groups were performed using Student’s independent *t*-test and the chi-square test. The correlations among patient characteristics were analyzed by Pearson correlation. Statistical significance was set at *p* < 0.05.

## 3. Results

At the last follow-up, none of the 26 patients required amputation or graft removal. The overall limb salvage rate was 100%. Four patients died without any reconstruction failure. Three patients died of pulmonary metastases. A total of 52 host-donor junctions were included in the analysis, divided in metaphyseal (*n* = 17) and diaphyseal junctions (*n* = 35). Primary union could be identified in all metaphyseal junctions, whereas in diaphyseal junctions 5 did not heal initially (5 of 35, 85.7% healing rate) (*p* = 0.255). The primary union rates in the vascularized and non-vascularized fibular graft groups were 82.1% and 100%, respectively (*p* = 0.088). Postoperative complications occurred in four of the 26 patients (15.4%), and included nonunion, infection, and plate fracture. All four patients with complications underwent reconstruction with a vascularized fibular graft. However, there was no statistical difference in the complication rate between the vascularized and non-vascularized fibular graft groups (28.6% vs. 0, respectively, *p* = 0.100). Two patients experienced both proximal and distal nonunions, and both received an additional autogenous iliac crest graft. The junction sites healed well after the reoperation. In addition, one of the two patients also developed postoperative graft infection, which was successfully treated with debridement. Bone healing was observed in one patient who had only distal nonunion in the tibia after the second operation with an autogenous iliac crest graft. In addition, one patient experienced a plate fracture, and the damaged plate was replaced (Table 2 and Table 3).

### 3.1. Upper Extremity Reconstruction Results

**Figure 3:**
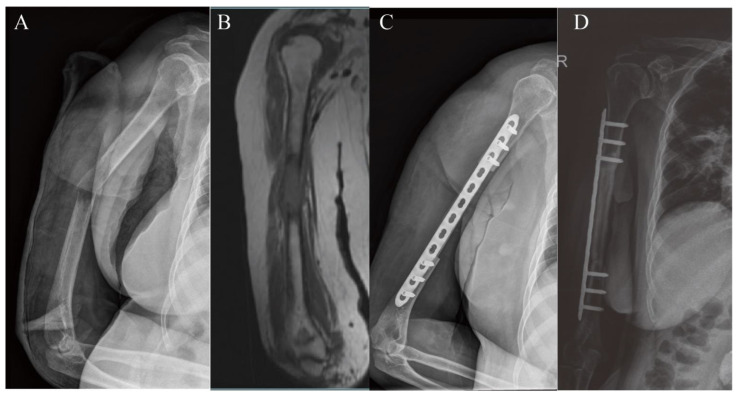
A patient underwent humerus diaphyseal reconstruction. (**A**,**B**) A female patient, aged 61 years, was histologically diagnosed with bone metastasis of thyroid carcinoma located in the right humerus. The tumor was examined by both X-ray and magnetic resonance imaging. (**C**) Photograph showing reconstruction with the fibular graft inserted into the medullary canal of the autograft and fixation with a single plate. (**D**) Radiograph showing primary union five months after surgery.

The mean follow-up duration was 65.8 ± 18.6 months. All patients underwent fibular graft reconstruction, and the combined biological reconstruction graft was an auto-inactivated tumor bone (*n* = 5) and allograft (*n* = 3) (Figure 3). Donor and recipient areas were located on the same side of the body. The mean length of resection was 89.4 ± 15.5 mm (70–120 mm). The average length of the fibular graft was 117.5 ± 12.8 mm (100–140 mm). The mean length of overlap between the fibular graft and recipient site was 28.1 ± 4.6 mm (20–35 mm). Only one patient received vascularized fibular graft transplantation. In this case, a vascularized fibular graft was used as a secondary procedure after the failure of a previous implant. The mean operation length was 160.6 ± 53.2 min (105–280 min) with a mean blood loss of 543.8 ± 134.8 mL. All the patients were treated with a single plate for internal fixation. Primary osseous union was achieved in 87.5% of the osteotomy sites. Bony union occurred in 87.5% of patients with a mean time of 4.6 ± 1.6 months (3–7 months). The complication rate in the upper extremity was 12.5%. The last follow-up results showed a high average MSTS score (28.1 ± 3.1 (range 21–30) out of 30) (Table 2 and Table 3).

### 3.2. Lower Extremity Reconstruction Results

**Figure 4:**
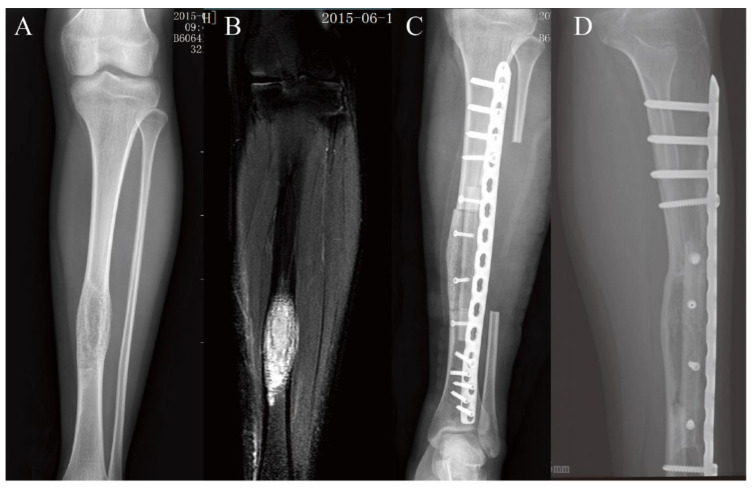
A patient received biological reconstruction in the left tibia. (**A**,**B**) A female patient, aged 19 years, was histologically diagnosed with adamantinoma. The tumor was assessed by both X-ray and magnetic resonance imaging. (**C**) Photograph showing reconstruction with the fibular graft inserted into the medullary canal of the autograft and fixation with a single plate. (**D**) Radiograph showing primary union five months after surgery.

**Figure 5:**
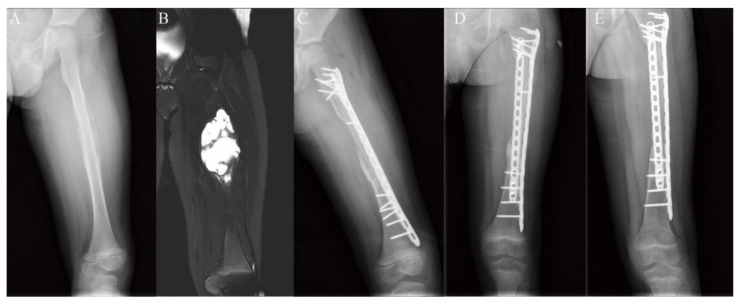
Diaphyseal reconstruction of the left femur. (**A**,**B**) A male patient, aged 8 years, was histologically diagnosed with Ewing’s sarcoma. The tumor was assessed by both radiograph and magnetic resonance imaging. (**C**) Photograph illustrating reconstruction with the fibular graft inserted into the medullary canal of the autograft and fixation with a single plate. (**D**) Photograph showing the replacement of the broken steel plate with double plates two months after surgery. (**E**) Radiograph showing primary union eight months after surgery.

The mean follow-up period was 77.3 ± 26.4 months. Sixteen of the 18 patients underwent intercalary resection and reconstruction with a fibular graft and an extracorporeal devitalized autograft. The allografts were applied in the other two cases as biological reconstruction grafts assembled with the fibula (Figure 4 and Figure 5). Only one fibular graft was harvested from the opposite limb of the recipient bone, because the ipsilateral fibula was removed. The mean defect, fibular graft, and graft overlap length were 117.2 ± 25.7 mm (70–170 mm), 152.1 ± 24.3 mm (130–226 mm), and 34.9 ± 10.9 mm (20–60 mm), respectively. Of the 18 patients, only five fibulas were applied as non-vascularized grafts. The average operation duration was 266.4 ± 115 min (90–495 min). The mean blood loss during the operation was 755.6 ± 463.6 mL (300–1500 mL). For tibial reconstruction, a single plate was used for internal fixation. Five of the six femoral reconstructions were fixed using double plates. The patient with a single plate experienced plate fracture in the early stage and achieved good results after replacement of the double plates for internal fixation. Radiographic examination showed a primary bony union in 16 patients after a mean follow-up of 6.9 months (3–11 months). Primary osseous union was observed in 91.7% of junctions. Complications occurred in three of the 18 patients who underwent lower extremity reconstruction: two patients (11.1%) developed nonunion and one experienced plate fracture (5.6%). The mean MSTS score was 26.7 ± 3.3 (range 19–30), with a mean MSTS rating of 89%. Please refer to Table 2 and Table 3.

## 4. Discussion

Due to the improvement of surgical reconstruction techniques, limb salvage surgery has become more common in the treatment of bone tumors of the extremities and is currently applied in 80% of patients [7,13]. Progress in endoprosthetic and biological design has led to a variety of reconstruction options after bone tumor resection. Reconstruction with artificial prostheses may produce good structural stability and functional recovery within a short period. However, the lower long-term graft survival rate of the prostheses is a major concern when applied in young patients [7,14]. The survival of the prostheses was 68% at 10 years in Hanna’s series [15]. Shehadeh et al. found that the survival curve of the prostheses showed a constant decline over time and the implant survival was 72% at 10 years and 37% at 20 years [16]. Implant failure is closely related to mechanical complications of the prosthesis, such as aseptic loosening [16]. Compared to prostheses, the biological grafts show relatively high long-term survival rates. Zekry et al. reported that the ten-year survival rate of the frozen autografts was 91.2% [17]. The overall graft survival in Campanacci’s study was 94.4% at five, ten, and 15 years [18]. Therefore, biological reconstruction can be considered as a more suitable option for long-term function and durable fixation. In our study, removal of graft or amputation was not recorded.

Since the free-vascularized fibular graft was first reported for the treatment of bone defects after resection of bone tumors, it has been widely used in the reconstruction of bone defects because of its abundant source of the bone, long vascularized graft, and acceptable donor site complications [4,8,12,19,20]. Considering its diameter and mechanical characteristics, fibular grafts are recommended for areas of lighter stress loads, such as the upper limb, mid-shaft tibia, and pediatric patients. Repo et al. [21] identified 20 patients who underwent reconstruction with a vascularized fibula graft in the upper extremity and found that the long-term outcomes of reconstruction with the free vascularized fibula graft were encouraging. Previous studies showed that the fibular graft performed better in the lower extremity reconstruction when combined with other stable biological graft, such as allograft and autoinactivated tumor bone. Errani et al. [13] examined a series of reconstructions with a vascularized fibula and massive bone allografts and showed that the overall limb salvage rate was 94% and the complication rate was 30% lower than that in reconstruction with massive bone allograft alone. Lu et al. [22] noted a lower risk of nonunion and infection in patients undergoing reconstruction with a vascularized fibular graft with frozen tumor-bearing autografts or Capanna reconstruction. Since the longer operation duration of composite reconstruction and the disadvantage of donor site complications, researchers discussed whether the allograft or autoinactivated tumor bone should be assembled with the vascularized fibula graft. Previous studies have shown that the complication rate for reconstruction with allograft or autograft alone was 27–57%, with a postoperative fracture rate of 6–45% [7,23,24,25,26,27,28]. The complication rate of composite reconstruction with a fibular graft was 30–48%, with a postoperative fracture rate of 0–44% [13,27,28,29,30]. Errani et al. concluded that there was no statistical difference between the two reconstruction methods in complication rates [27]. However, fracture may be less likely in composite reconstruction, as fibular hypertrophy can play a role in compensation by creeping substitution [7,28]. Thus, composite reconstruction seems to be a better option for the satisfied results and faces a longer surgical time and technical challenges [11]. This study also showed satisfactory MSTS scores in patients who underwent reconstruction with frozen tumor-bearing autografts and vascularized fibular grafts, comparable to those in patients who underwent Capanna reconstruction. Therefore, when combined with a fibular graft, frozen tumor-bearing autografts can be used as an alternative to allografts [22]. In our series, both devitalized autografts and allografts were used to reconstruct the bone defects.

Vascularized fibular grafts have been well accepted by orthopedic surgeons for defect reconstruction. However, vascularized and non-vascularized fibular grafts have advantages and disadvantages [31,32]. Lenze et al. [32] reviewed 36 patients who were treated with non-vascularized fibular grafts and noted that non-vascularized fibula reconstruction achieved encouraging outcomes in union (94%) and hypertrophy (85%) at graft-host junctions with good remodeling capacity at the donor site. The non-vascularized fibular graft can shorten the length of surgery with no significant difference in long-term outcomes [10,28]. A shorter union time was observed in the vascularized group because of its biological activity [33]. This graft should be used especially for the reconstruction of massive segmental defects and may be a better option when local blood supply is poor. Schuh et al. [34] followed 53 patients who had vascularized (49%) and non-vascularized (51%) fibular autografts and found no statistically significant difference in functional or radiological results between the two groups. In this study, most humeral reconstruction cases (87.5%) were treated with a non-vascularized fibular graft. In contrast, in the lower extremities, the vascularized graft had a higher utilization rate (72.2%). This is because the lower extremity suffers more force than the upper extremity. The vascularized fibula may ensure the survival rate of lower extremity grafts. Although the administration of vascularized or non-vascularized grafts did not significantly influence blood loss (*p* = 0.189), union time (*p* = 0.875), or functional outcome (*p* = 0.501), a statistically significant correlation between operation time and the vascularized or non-vascularized method (*p* = 0.012) was observed in lower extremity reconstructions (Table 4). The comparison between the vascularized and non-vascularized graft groups was not analyzed in the upper extremity reconstruction since only one patient received a vascularized fibular graft. There was no statistical difference in union rate (82.1% vs. 100%, *p* = 0.088) and complication rate (28.6% vs. 0, *p* = 0.100) between the vascularized and non-vascularized fibular graft groups (Table 3). Since the length of the resection would have been a confounding factor, we analyzed the difference in resection length between the vascularized and non-vascularized fibula group. The result showed that the resection length in vascularized and non-vascularized fibula group was 121.1 ± 25.7 mm and 94.2 ± 19.0 mm, respectively (*p* = 0.006). Besides, correlation analysis between resection length and each factor was conducted. The results indicated that operation duration (*p* = 0.001), blood loss (*p* < 0.001) and time to union (*p* = 0.002) had a significantly positive relation with resection length, but that MSTS score (*p* = 0.010) was negatively correlated to resection length. The results showed that the resection length affected the outcome of reconstruction and negatively correlated with good results. There was no significant difference in reconstruction results between the vascularized and non-vascularized fibula groups, but the resection length in the vascularized group was longer than that in the non-vascularized group. This suggested that the advantage of vascularized grafts may be overshadowed by the resection length. Lenze et al. [32] concluded that non-vascularized fibula reconstruction was a valuable treatment option for patients with segmental defects of less than 12 cm. In our series, the resection length in the non-vascularized fibula group was 94.2 ± 19.0 mm (range 70–140 mm), and non-vascularized fibula reconstruction showed good outcomes comparable to vascularized fibula reconstruction. It seems that non-vascularized fibula reconstruction is still a promising treatment option for relatively long resection lengths.

Fibular grafts are routinely combined with other biological grafts when applied to lower extremity reconstructions. The cases in our study also underwent this procedure. When combined with a fibular graft, devitalized autograft (21 of 26) was the most common biological graft in our series. Our salvage rate was 100%, which is comparable to rates of 82% to 100% reported in previous studies [13,35,36]. The use of fibular grafts with autografts or allografts in reconstruction after diaphyseal bone tumor resection has been reported to lead to postoperative complications, such as nonunion, infection, and fracture. In this series, 4 of the 26 patients developed five postoperative complications, including nonunion (*n* = 3), infection (*n* = 1), and plate fracture (*n* = 1). None of the complications recorded in our study threatened graft survival after reoperation. The nonunion rate of allografts with vascularized fibular grafts ranged from 0% to 33% in previous studies [28], and the nonunion rate was 11.5% in our series. In addition, previous studies have shown that metaphyseal junctions heal more easily [37,38]. In this series, union rates in the metaphyseal and diaphyseal junctions were 100% and 85.7%, respectively. However, there was no statistically significant difference between the two groups (*p* = 0.255). All nonunions occurred at the diaphyseal junctions and were treated well after local bone grafting using an iliac crest allograft. Thus, patients with nonunion can achieve good results through local bone grafting. A single plate was used as the internal fixation method for the upper extremity reconstruction. One patient who had been treated with a single plate experienced plate fracture and achieved good results after replacement of the double plates in the femoral reconstruction. Thus, we suggest that double plates should be used for lower extremity reconstruction. A prospective cohort study should be conducted in subsequent clinical studies to verify this suggestion. A shorter union time and a higher MSTS score were observed in our results compared with those reported in previous studies [39,40] (Table 3). In summary, the surgical procedure introduced above provides an acceptable option for reconstructing diaphyseal bone tumor resections.

The main limitations of this study are its retrospective design, small sample size, and lack of a control group. And using the MSTS score might have limitations, since the MSTS score is highly affected by emotional acceptance and daily satisfaction. Therefore, the actual function might not be reflected by the MSTS scores. Fortunately, the outcome of vascularized and non-vascularized fibular grafts has been preliminarily described. Osseous oncologic reconstruction with a composite free fibula inside other biological grafts seemed to provide better radiological and functional results.

## 5. Conclusions

The use of a composite free fibula inside other biological grafts provides a reasonable and durable option for extremity osseous oncologic reconstruction, with an acceptable complication rate and good functional results. Nonunion mainly occurs in the diaphyseal junctions and can be treated with local bone grafting. There was no obvious difference in blood loss, union time, or functional outcome between the vascularized and non-vascularized fibular grafts in lower extremity reconstruction. This may be related to the influence of the resection length on the choice of reconstruction method. Compared with a vascularized fibular graft, a shorter operation length can be obtained with a non-vascularized fibular graft. The two plates used as internal fixation methods are recommended for lower extremity reconstruction, especially in the femur. The interesting outcomes verified in this study show the importance of further comparison studies with larger sample sizes to other reconstructive methods.

## Figures and Tables

**Table 1:** Demographic Data of the 26 Patients.

Case	Gender	Age	Tumor Location	Side	Diagnosis
1	Female	27	Humerus	Right	BMTC
2	Female	61	Humerus	Right	BMTC
3	Female	17	Humerus	Right	OS
4	Male	56	Humerus	Right	BMRCC
5	Male	62	Humerus	Left	BMRCC
6	Male	46	Humerus	Right	CS
7	Female	37	Humerus	Left	BMTC
8	Male	42	Humerus	Left	BMRCC
9	Male	37	Tibia	Left	OS
10	Male	64	Tibia	Right	BMBC
11	Female	48	Tibia	Right	AD
12	Female	19	Tibia	Left	AD
13	Female	63	Tibia	Right	HB
14	Female	51	Tibia	Right	OD
15	Female	39	Tibia	Left	OS
16	Female	28	Tibia	Left	AD
17	Male	61	Tibia	Right	BMRCC
18	Female	51	Tibia	Right	OD
19	Female	45	Tibia	Right	OS
20	Male	58	Tibia	Left	BMBC
21	Male	43	Femur	Left	OD
22	Female	56	Femur	Left	PO
23	Male	18	Femur	Right	OS
24	Male	8	Femur	Left	ES
25	Female	40	Femur	Left	ES
26	Male	16	Femur	Left	OS

BMTC, bone metastasis of thyroid carcinoma; BMRCC, bone metastasis of renal cell cancer; CS, chondrosarcoma; OS, osteosarcoma; BMBC, bone metastasis of bladder cancer; AD, adamantinoma; HB, hemangioma of bone; OD, osteofibrous dysplasia; PO, parosteal osteosarcoma; ES, Ewing’s sarcoma.

**Table 2:** Operative Details and Follow-up.

Case	Resection Length (mm)	Fibula Length (mm)	Vascularized Fibula (Yes/No)	Type of Reconstruction	Operation Duration (min)	Blood Loss (mL)	Follow-Up (Month)	Time to Union (Month)	MSTS Score	Complications
1	120	140	No	devitalized autograft, single plate	150	600	51	3	30	/
2	85	110	No	allograft, single plate	150	500	62	5	29	/
3	70	100	No	allograft, single plate	150	400	96	3	30	/
4	90	120	Yes	devitalized autograft, single plate	105	500	37	/	21	Infection, Nonunion
5	100	130	No	allograft, single plate	180	500	57	7	28	/
6	90	120	No	devitalized autograft, single plate	280	850	83	6	30	/
7	75	110	No	devitalized autograft, single plate	120	500	74	3	30	/
8	85	110	No	devitalized autograft, single plate	150	500	66	5	27	/
9	115	150	Yes	devitalized autograft, single plate	247	1000	42	6	29	/
10	120	150	Yes	devitalized autograft, single plate	180	600	69	8	27	/
11	110	150	Yes	devitalized autograft, single plate	180	300	59	6	30	/
12	95	130	No	devitalized autograft, single plate	90	500	72	5	30	/
13	155	180	Yes	devitalized autograft, single plate	300	850	94	/	23	Nonunion
14	110	147	Yes	devitalized autograft, single plate	240	300	89	6	28	/
15	115	150	Yes	devitalized autograft, single plate	240	300	123	5	22	/
16	70	130	Yes	devitalized autograft, single plate	240	300	73	3	30	/
17	90	130	No	devitalized autograft, single plate	158	500	82	8	24	/
18	90	130	No	devitalized autograft, single plate	180	300	79	6	28	/
19	120	150	Yes	devitalized autograft, single plate	240	500	74	6	28	/
20	90	130	No	devitalized autograft, single plate	235	300	38	7	27	/
21	140	170	No	allograft, double plate	150	1000	124	9	29	/
22	110	130	Yes	devitalized autograft, double plate	495	1000	110	11	28	/
23	170	226	Yes	devitalized autograft, double plate	480	1450	38	10	19	/
24	130	150	Yes	devitalized autograft, single plate	420	1500	58	8	27	Plate fracture
25	150	170	Yes	allograft, double plate	330	1450	104	/	22	Nonunion
26	130	165	Yes	devitalized autograft, double plate	390	1450	64	6	30	/

**Table 3:** Summary of Operative Details and Outcome.

	Follow-Up (Month)	Resection Length (mm)	Fibula Length (mm)	Overlap Length (mm)	Operation Duration (min)	Blood Loss (mL)	Time to Union (Month)	MSTS Score	Bony Union Rate	Incidence of Complication
Upper extremity	65.8 ± 18.6	89.4 ± 15.5	117.5 ± 12.8	28.1 ± 4.6	160.6 ± 53.2	543.8 ± 134.8	4.6 ± 1.6	28.1 ± 3.1	87.5%	12.5%
Lower extremity	77.3 ± 26.4	117.2 ± 25.7	152.1 ± 24.3	34.9 ± 10.9	266.4 ± 115	755.6 ± 463.6	6.9 ± 2	26.7 ± 3.3	91.7%	16.7%
Total	73.8 ± 24.5	108.7 ± 26.3	141.5 ± 26.7	32.8 ± 9.8	233.8 ± 110.8	690.4 ± 401.5	6.2 ± 2.2	27.2 ± 3.2	88.5%	15.4%
Vascularized graft	73.9 ± 27.1	121.1 ± 25.7	154.9 ± 25.8	33.8 ± 12.2	291.9 ± 116.7	821.4 ± 484.7	6.8 ± 2.3	26.0 ± 3.8	82.1%	28.6%
Non-vascularized graft	73.7 ± 22.3	94.2 ± 19.0	125.8 ± 18.3	31.7 ± 6.5	166.1 ± 49.9	537.5 ± 203.5	5.6 ± 2.0	28.5 ± 1.8	100%	0
Metaphyseal junctions	/	/	/	/	/	/	/	/	100%	/
Diaphyseal junctions	/	/	/	/	/	/	/	/	85.7%	/

**Table 4:** Results According to the Management of the Fibular Graft and Comparison of the Lower Extremity Reconstruction.

	Upper Extremity		Lower Extremity		
	Vascularized Graft	Non-Vascularized Graft	Vascularized Graft	Non-Vascularized Graft	*p*-Value
Operation duration (min)	105 *	168.6 ± 52.1	306.3 ± 107.7	162.6 ± 52.4	0.012
Bleeding volume (ml)	500 *	550 ± 144.3	846.2 ± 495.2	520 ± 286.4	0.189
Time to union (month)	/	4.6 ± 1.6	6.8 ± 2.3	7 ± 1.6	0.875
MSTS Score	21 *	29.1 ± 1.2	26.4 ± 3.6	27.6 ± 2.3	0.501

* Only one patient underwent reconstruction with a vascularized graft in the upper extremity and primary osseous union was not observed after the first surgery.

## Data Availability

The data of this study are available from the corresponding author upon reasonable request.
